# Weakened effective connectivity between salience network and default mode network during resting state in adolescent depression

**DOI:** 10.3389/fpsyt.2024.1386984

**Published:** 2024-04-04

**Authors:** David Willinger, Isabelle Häberling, Iva Ilioska, Gregor Berger, Susanne Walitza, Silvia Brem

**Affiliations:** ^1^ Department of Child and Adolescent Psychiatry and Psychotherapy, University Hospital of Psychiatry Zurich, University of Zurich, Zurich, Switzerland; ^2^ Neuroscience Center Zurich, University of Zurich and ETH Zurich, Zurich, Switzerland; ^3^ Department of Psychology and Psychodynamics, Karl Landsteiner University of Health Sciences, Krems an der Donau, Austria; ^4^ Department of Cognitive Neuroscience, Donders Institute for Brain, Cognition and Behaviour, Radboud University Medical Centre, Nijmegen, Netherlands

**Keywords:** adolescence, affective disorders, brain connectivity, resting-state fMRI, SSRI

## Abstract

Adolescent major depressive disorder (MDD) is associated with altered resting-state connectivity between the default mode network (DMN) and the salience network (SN), which are involved in self-referential processing and detecting and filtering salient stimuli, respectively. Using spectral dynamical causal modelling, we investigated the effective connectivity and input sensitivity between key nodes of these networks in 30 adolescents with MDD and 32 healthy controls while undergoing resting-state fMRI. We found that the DMN received weaker inhibition from the SN and that the medial prefrontal cortex and the anterior cingulate cortex showed reduced self-inhibition in MDD, making them more prone to external influences. Moreover, we found that selective serotonin reuptake inhibitor (SSRI) intake was associated with decreased and increased self-inhibition of the SN and DMN, respectively, in patients. Our findings suggest that adolescent MDD is characterized by a hierarchical imbalance between the DMN and the SN, which could affect the integration of emotional and self-related information. We propose that SSRIs may help restore network function by modulating excitatory/inhibitory balance in the DMN and the SN. Our study highlights the potential of prefrontal-amygdala interactions as a biomarker and a therapeutic target for adolescent depression.

## 1 Introduction

Major depressive disorder (MDD) is a common and debilitating condition with onset peaking in adolescence (1) which is estimated to have a lifetime prevalence of approximately 11% (2). Adolescent MDD exerts detrimental effects on physical and mental health, impairing academic, occupational, and social functioning. Additionally, it elevates the risk of recurrent MDD episodes in adulthood, co-occurring psychiatric and medical conditions, such as anxiety disorders, and suicide, the second leading cause of mortality in individuals aged 15 to 19 years (3, 4). The underlying neurobiological factors of the emergence and early trajectory of MDD in adolescence remain poorly understood, despite the known adverse consequences of this disorder.

Previous studies have reported several changes of large-scale functional brain networks in adult and adolescent depression. Large-scale networks are defined as a distributed set of brain regions that show a temporal correlation during a task or spontaneous thought (i.e., rest (5)). They are thought to support embedding predictions and prediction errors which dynamically adjust the brain’s internal generative models based on sensory inputs and prior expectations (6, 7). These models have a hierarchical structure, meaning that higher-level processing regions generate predictions that are sent to lower-level regions, where they are compared with the incoming sensory data. The prediction error – the mismatch between predictions and data – is propagated back to the higher-level regions to update the models (7). During rest, it has been proposed that the dynamic fluctuations quantified as connectivity in large-scale networks represent an optimization of generative models for future interactions (8).

Multiple studies found that connectivity of the default mode network (DMN) is affected in MDD. Core nodes of the DMN, the medial prefrontal cortex (MPFC) and posterior cingulate cortex (PCC) have shown alterations in children and adults with or at risk for MDD (9–14). A recent longitudinal study linked altered developmental trajectory of DMN connectivity to depressive symptoms in youth (15), indicating that clinically relevant alterations manifest relatively early in brain development. In addition, there is compelling evidence that core nodes of the affective network (the amygdala) and the ventral attention network [dorsal anterior cingulate cortex, ACC (16)] – together forming the salience network [SN (17)] – show aberrant connectivity in adolescent (11, 18–22) and adult MDD (13, 14).

These alterations in large-scale networks are intriguingly aligned with the concepts of predictive processing and their potential role in the aetiology of depressive symptoms. It has been assumed that those network changes represent the neural manifestation of the predictive biases and altered perception characteristic of depression, further connecting the theoretical framework with the observed neurobiology (23, 24). As individuals with MDD tend to anticipate negative events more frequently than positive ones, the connectivity changes of core nodes of the DMN become crucial. The DMN’s involvement in self-referential processing and its role in maintaining the most abstract predictions of the internal models could contribute to the formation of these biased predictions (24–26). The SN’s role in regulating attention (27) and encoding the relevance of both external and internal stimuli could in turn influence the selection of which sensory prediction errors to attend to by modulating the gain on prediction error signals orginating from the sensory periphery (28). Additionally, aberrant connectivity within core nodes of the SN might amplify the attentional focus on (negative) prediction errors. This could lead to a vicious cycle where heightened sensitivity to negative information reinforces maladaptive perception.

Furthermore, the intricate interactions between these large-scale networks, as proposed by the triple-network model (29), might be pivotal in understanding the emergence and persistence of depressive symptomatology. Altered connectivity between these networks, not only underlie maladaptive self-referential processing and emotional regulation but also hinder the brain’s ability to effectively update its internal model and adapt to external cues. This insensitivity to external cues, driven by skewed predictions and impaired network communication, likely contributes to the cognitive and emotional symptoms commonly observed in depression (24, 30). More specifically, altered amygdala function has been suggested to contribute to maladaptive weighting of relevance (i.e., loss of precision or heightened uncertainty about relevance) of incoming bottom-up signals in depression (19, 24, 28, 31). The resulting imprecision of bottom-up signals may entail failure of updating the internal model and their dismissal which may underlie symptoms such as rumination (24). In light of this, there is evidence of reduced connectivity between the amygdala and other SN regions in adolescent depression (32) which could indicate impaired detection and integration of relevant sensory signals that challenge the models’ prediction. Altered connectivity between DMN and SN (e.g., the dorsal ACC, 16) could in turn be interpreted as altered precision over the predictions of the internal model, contributing to a “locked-in state” of negative thoughts (28). Altogether, current evidence suggests that the interactions between intrinsic brain networks, the DMN and the SN, might be closely linked to depression and contribute to the cardinal symptoms of rumination and negative mood.

The goal of the current study was to examine the functional integration of DMN and SN in adolescent depression. We used spectral dynamic causal modelling (spDCM; (33)) in the Parametric Empirical Bayes (PEB) framework to study the effective connectivity of the DMN and the important nodes of the SN during rest using multi-echo fMRI. Spectral DCM allows to model the directed relationships between brain networks and determines regions that are driving activity in other regions and their respective input sensitivity or excitatory-inhibitory balance (i.e., interregional self-inhibition or synaptic gain). In the predictive coding framework the excitatory-inhibitory balance reflects the precision of prediction errors encoded in the excitability of superficial pyramidal cells that is affected by both classical neuromodulators and inhibitory interneurons – lending the self-inhibition parameter to a straight-forward interpretation in terms of efficiency of information processing and network synchrony (i.e., a higher the self-inhibition reduces the influence of other regions) (34).

We investigated connectivity between the MPFC and PCC comprising the principal nodes of the DMN, and the dorsal ACC and bilateral amygdalae as part of the SN. The primary hypothesis of our study was that the effective resting-state connectivity between the amygdalae and the default mode network is altered in adolescents with MDD (14, 19). In addition, we hypothesised that the amygdalae show hypoconnectivity within the SN (32). Finally, we expected a decrease in the self-inhibition parameters of the spDCM, which regulate the excitatory-inhibitory balance of the regions. Such a decrease would lead to more excitability within the DMN regions, indicating aberrant encoding of precision (24, 33, 35).

## 2 Materials and methods

### 2.1 Participants

Thirty MDD patients and 32 healthy individuals matched for age, IQ, sex, and handedness participated in this study (**Table 1**). To assess the participants, a semistructured clinical interview was administered using either the Schedule for Affective Disorders and Schizophrenia for School-Age Children–Present and Lifetime Version (Kiddie-SADS, 36) or the Mini-International Neuropsychiatric Interview for Children and Adolescents (MINI-KID, 37). Criteria for the diagnosis of MDD in accordance with both the International Classification of Diseases (ICD-10) and the Diagnostic and Statistical Manual of Mental Disorders (DSM-5) were met by all patients, as determined by the Kiddie-SADS or MINI-KID, respectively. Inclusion criteria for study participation encompassed individuals within the age range of 8 to 18 years. Exclusion criteria encompassed any contraindication for magnetic resonance imaging (MRI) and, for the control group, the absence of any prevailing psychiatric axis-1 diagnosis. All participants gave their written informed consent and were financially reimbursed at the end of the study. Patients received psychotherapy as needed during the study. This study was approved by the ethics committee of the Canton of Zurich and was conducted in accordance with the Declaration of Helsinki.

**Table 1:** Clinical and demographic characteristics of study participants.

	Controls	MDD	Test statistic	p value^a^
Age (years), range (min-max)	16.2 (1.9), 11.2-18.8	16.1 (1.4), 12.8-18.7	U=553.5	.425
Sex (males), No. (%)	10 (30%)	10 (33%)	χ^2^(1)=0.07	.796
Handedness (right), No. (%)	32 (97%)	28 (93%)	χ^2^(1)=0.46	.500
In-scanner movement (FD, mm)	0.16 (0.06)	0.17 (0.06)	t(61)=0.69	0.492
CD-RISC	72.9 (10.1)	38.6 (15.6)	t(58)=10.16	<.001
CDI	8.4 (6.6)	29.6 (9.3)	U=38.0	<.001
Anhedonia	2.3 (2.2)	10.5 (2.8)	U=13.5	<.001
Negative mood	2.2 (2.0)	6.4 (2.4)	U=88.0	<.001
Negative self-esteem	1.0 (1.2)	5.0 (1.7)	U=42.0	<.001
Ineffectiveness	1.2 (1.2)	5.0 (1.9)	U=54.5	<.001
Interpersonal problems	1.1 (1.2)	3.7 (1.5)	U=74.5	<.001
Stomach	0.6 (0.6)	1.1 (0.8)	U=301.5	.018
RIAS IQ	104.5 (6.9)	108.0 (8.7)	t(60)=-1.75	.079
PSS	22.4 (6.6)	28.8 (7.7)	t(57)=-3.44	.001
SDQ	8.8 (5.3)	16.3 (5.6)	t(56)=-5.26	<.001
WISC-IV Digitspan (forward)	8.9 (2.1)	8.8 (2.0)	t(60)=0.32	.747
WISC-IV Digitspan (backward)	8.6 (1.6)	9.4 (2.0)	t(60)=-1.70	.094
WISC-IV Mosaic	57.0 (5.7)	59.0 (6.2)	t(56)=-1.27	.208
Current Medication, No. (%)				
No medication	NA	10 (33%)	NA	NA
SSRI	NA	18 (60%)	NA	NA
Dual-action antidepressant^b^	NA	2 (7%)	NA	NA
NERI	NA	2 (7%)	NA	NA
Antipsychotic^c^	NA	2 (7%)	NA	NA
Methylphenidate	NA	2 (7%)	NA	NA

Data are presented as mean (SD) if not indicated otherwise.

CD-RISC, Connor-Davidson Resilience Scale; CDI, Children Depression Inventory; FD, framewise displacement; RIAS, Reynolds Intellectual Assessment Scales; PSS, Perceived Stress Scale; SDQ-K, Strength and Difficulty Questionnaire for Children; WISC, Wechsler Intelligence Scale for Children.

a Uncorrected p values for between-group comparisons; significance threshold p<.05.

b Serotonin-noradrenalin reuptake inhibitor.

c Used for behavioral control.

### 2.2 Imaging and preprocessing

Image data acquisition was conducted on an Achieva 3T scanner (Philips Medical Systems, Best, the Netherlands) using a 32-channel head coil array. We acquired a T1-weighted structural scan of each subject [MP-RAGE, aligned at AC-PC, flip angle = 9°, voxel size = 1.05 × 1.05 × 1.2*mm* ^3^, field of view = 270 × 253*mm* ^2^, 170 sagittal slices]. Subsequently, T2*-weighted images were acquired using a multi-echo multi-slice echo-planar images sequence [200 volumes per session, *TR* = 2300*ms*, *TE* = 13,31,49*ms*, 33 slices, voxel size = 3.75 × 3.75 × 3.79*mm* ^3^, matrix size = 64 × 64*px*, flip angle = 80°, gap = 0.39*mm*, SENSE-factor = 2, MB-factor = 2] during a ~6 minute resting state with eyes open. During preprocessing, the volumes corresponding to the three echoes were separately despiked (spmup_despike.m, https://github.com/CPernet/spmup/wiki/spmup_despike.m) and slice-time corrected using SPM12. The motion parameters were calculated from the first echo and applied to the remaining echoes using mcFLIRT from the FSL toolbox (38). TEDANA, that is part of the Multi Echo Independent Component Analysis (MEICA) package [https://afni.nimh.nih.gov/pub/dist/src/pkundu/meica.py (39)], was used to perform state-of-the-art TE-dependent ICA-based denoising and T2* weighted averaging of optimally combined echoes and fully leverage all available data – particularly in ventral regions (40). The denoised images were coregistered to the structural scan and normalized to the Montreal Neurological Institute (MNI)-152 template space using the deformation fields derived from segmentation. Finally, we applied spatial smoothing using a 6*mm* full-width-half-maximum kernel to the functional images. Subsequently, a general linear model was created including the motion parameters and discrete cosine transform for band-pass-filtering (frequency range 0.08–0.01 Hz). An inspection of the mean framewise displacement (FD; 41) in patients and controls showed no evidence of differences in head motion between groups, *t* (60)= 0.003, *p* = .99, no individual subject showed a mean FD in excess of 0.27*mm*.

### 2.3 Timeseries extraction and statistical analysis

The coordinates for extraction of regional signals for the spDCM analysis were based on the literature (42, 43). We created spherical search volumes (*r*=8mm) for the network nodes of the MPFC (x=-1, y=54, z=27mm MNI) and the PCC (x=0, y=-52, z=7mm MNI), the dorsal ACC (x=0, y=21, z=36mm MNI), and the bilateral amygdalae (AMY; x= ± 19, y=-2, z=-21mm MNI). We centered the spherical ROI around each participant’s maximum within the search volume and extracted the first eigenvariate of the time course of active voxels (*p* <.05, uncorrected). Realignment parameters obtained during preprocessing were partialed out.

We applied spectral dynamic causal modelling (spDCM) to estimate intrinsic effective connectivity from resting state fMRI data (44). SpDCM is a method that models the cross-spectra of the blood oxygenation level dependency (BOLD) signals, which are a more comprehensive measure of connectivity than the conventional zero-lag correlation. SpDCM allows us to determine the directed connectivity strengths between brain regions that drive their activity, as well as their input sensitivity or synaptic gain, which corresponds to the excitatory/inhibitory balance of each region.

We set up a fully connected model on all interregional connections. The analysis was conducted within the PEB framework where the full DCM model was estimated in an empirical Bayesian inversion scheme for each participant (45). Group effects on the DCM parameters (i.e., connectivity strengths) were analysed with a second-level PEB model to find group differences between patients and controls within the specified brain network. We used a Bayesian model reduction procedure to discard the model parameters not contributing to the model evidence in a greedy-search. This procedure stops when it removes a connection that decreases the model evidence. We analyzed the average intrinsic connectivity with group as predictor and sex, age, and handedness as covariates. To investigate potential effects of selective serotonine reuptake inhibitor (SSRI) intake in the eighteen patients on SSRIs, we added an additional regressor for SSRI intake to the PEB model. One patient was excluded from this analysis due to not disclosing their medication status. Group-level parameters were determined by averaging the best 256 nested models, weighted by their posterior probability. Parameters were considered significant when exceeding a 95% posterior probability of being present, based on the model evidence. As a last step, to validate our results, we used leave-one-out cross-validation (LOOCV; *spm_dcm_loo.m*) and assessed the predictive validity of the individual parameters of the connectivity model. To this end, we used the list of class probabilities for each subject and used it to retrieve the Receiver Operating Characteristic (ROC) curve and the Area Under the Curve (AUC) – the probability of a correct classification – with 95% confidence bounds across the cross-validation runs.

## 3 Results

### 3.1 Demographics and clinical symptoms

Patients and controls did not differ significantly in age, sex, IQ, handedness, or in-scanner movement (*p* >.05). They differed in clinical symptom scales: patients scored significantly higher on the Child Depression Inventory (*p* <.001), Connor-Davidson Resilience Scale (*p* <.001), Perceived Stress Scale (*p* = .001) and the Strength and Difficulty Questionnaire (*p* <.001). In-scanner movement during the scan measured as framewise displacement did not differ between the groups (*p* = 0.492). Sample characteristics and test results are summarized in **Table 1**.

### 3.2 Spectral DCM model structure across groups

**Figure 1:**
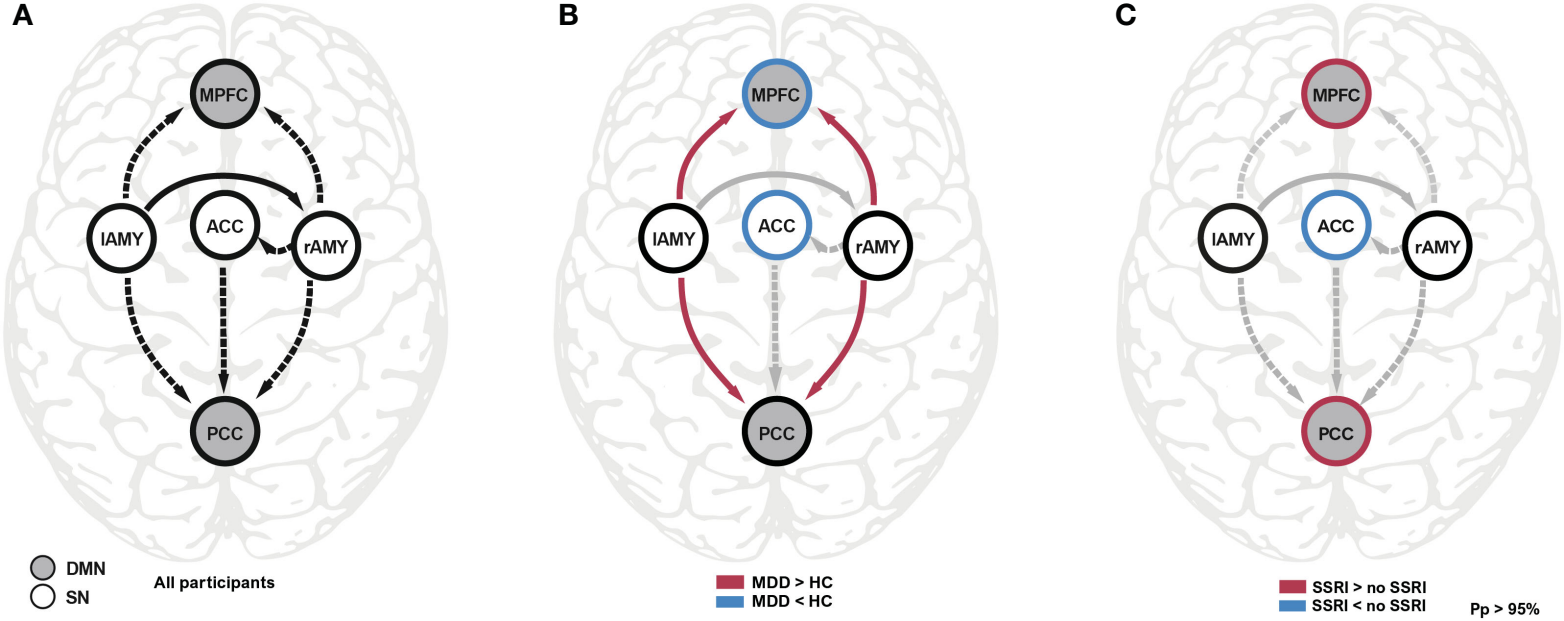
Spectral DCM analysis during resting state. **(A)** The common effect reflects the average connectivity and model structure across all participants. **(B)** Connectivity differences between controls and patients were found between amygdala and the core nodes of the DMN, as well as the self-inhibition parameters of MPFC and ACC. The latter regions were disinhibited (i.e. more sensitive to input) compared to healthy controls. **(C)** Patients taking SSRI showed decreased self-inhibition in the ACC and increased self-inhibition in the MPFC and PCC compared to patients not taking SSRIs. Detailled results are reported in **Table 2**. ACC, anterior cingulate cortex; DMN, default mode network; HC, healthy controls; lAMY, left amygdala; MDD, major depressive disorder; MPFC, medial prefrontal cortex; PCC, posterior cingulate cortex; Pp, posterior probability; SSRI, selective serotonin reuptake inhibitor; rAMY, right amygdala; SN, salience network.

The overall model structure across groups revealed by spDCM was primarily characterized by the directed negative coupling between SN and DMN (**Table 2**, **Figure 1A**). In particular, we found significant connectivity from the lAMY and PCC (expected value = −0.314 Hz, PP = 1.00) and MPFC (expected value = −0.234 Hz, PP = 1.00) and from rAMY to PCC (expected value = −0.425 Hz, PP = 1.00) and MPFC (expected value = −0.234 Hz, PP = 1.00). A unidirectional inhibitory connection from the ACC to the PCC portion of the DMN was also significant across both groups (expected value = −0.222 Hz, PP = 1.00). Connectivity within SN was characterized by functional coupling from the lAMY to rAMY (expected value = 0.234 Hz, PP = 1.00) and from the rAMY to the ACC (expected value = −0.149 Hz, PP = 1.00). Other connections were pruned from the model as they did not contribute significantly to the model evidence (**Table 2**, **Figure 1A**).

**Table 2:** Connectivity strength (posterior probability) during resting state obtained by Bayesian model averaging of PEB model parameters.

Connection type	Common	MDD	SSRI	Sex	Age	Handed.
Endogenous parameters						
PCC→lAMY	–	–	–	–	–	–
PCC→rAMY	–	–	–	–	–	–
PCC→MPFC	–	–	–	–	–	–
PCC→ACC	–	–	–	–	–	–
MPFC→PCC	–	–	–	-0.080	0.058 (1)	–
MPFC→ACC	–	–	–	–	–	–
MPFC→lAMY	–	–	–	–	–	–
MPFC→rAMY	–	–	–	–	–	–
ACC→PCC	-0.222 (1)	–	–	–	–	0.198 (1)
ACC→MPFC	–	–	–	–	–	–
ACC→lAMY	–	–	–	–	–	–
ACC→rAMY	–	–	–	–	–	–
lAMY→PCC	-0.314 (1)	0.111 (1)	–	-0.09	–	–
lAMY→MPFC	-0.229 (1)	0.098 (1)	–	–	-0.061	–
lAMY→ACC	–	–	–	–	–	–
lAMY→rAMY	0.234 (1)	–	–	–	–	–
rAMY→PCC	-0.425 (1)	0.167 (1)	–	–	–	–
rAMY→MPFC	-0.234 (1)	0.094 (1)	–	–	–	–
rAMY→ACC	-0.149 (1)	–	–	–	-0.062 (1)	–
rAMY→lAMY	–	–	–	–	–	–
Self-inhibition parameters						
lAMY→lAMY	0.603 (1)	–	–	–	–	–
rAMY→rAMY	0.760 (1)	–	–	–	0.047 (1)	–
ACC→ACC	0.134 (1)	-0.217 (1)	-0.175 (1)	–	–	–
MPFC→MPFC	0.331 (1)	-0.119 (1)	0.128 (1)	0.107	–	–
PCC→PCC	-0.280 (1)	–	0.166 (1)	–	–	–

Between-region connections are in units of Hz. Self-inhibition parameters, where the source and target are the same, are the log of scaling parameters that multiply up or down the default value −0.5Hz. Posterior probabilities are given in the brackets. *n* = 61. lAMY, left amygdala; rAMY, right amygdala; ACC, anterior cingulate cortex; MPFC, medial prefrontal cortex; PCC, posterior cingulate cortex.

“–” means “Pruned from the full model”.

### 3.3 Aberrant connectivity from amygdala to default mode network in depression

**Figure 2:**
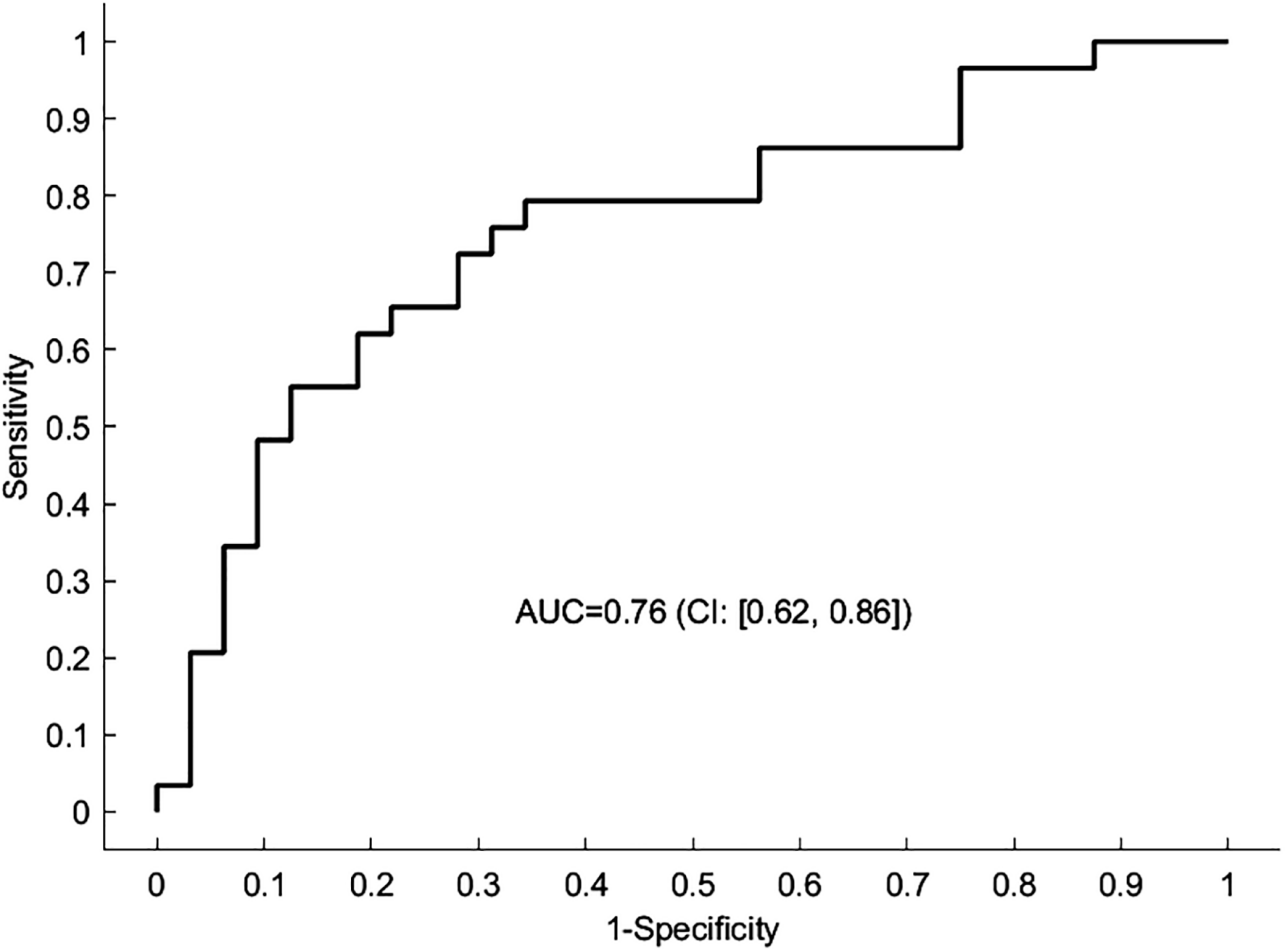
Predicting the diagnostic status using the spDCM connectivity parameters. The receiver operating characteristic (ROC) curve depicted here represents the outcome of a leave-one-out cross-validation procedure applied to the DCM analysis. The curve illustrates the trade-off between sensitivity and specificity for the predictive model across different thresholds. The area under the curve (AUC) serves as a statistical measure of the model’s ability to correctly classify a new participant as having MDD or not. An AUC of 1 indicates perfect predictive accuracy, whereas an AUC of 0.5 suggests no discriminative power, equivalent to random chance.

We found evidence that connectivity in patients is significantly altered compared to healthy controls. Most prominently, the connections between bilateral amygdalae and both nodes of the DMN were affected (**Figure 1B**). Participants with MDD exhibited weaker inhibitory (more positive) connectivity between lAMY and DMN (MPFC: expected value of group difference = 0.098 Hz, posterior probability, PP = 1.00; PCC: expected value of group difference = 0.111 Hz, PP = 1.00), and rAMY and DMN (MPFC: expected value of group difference = 0.094 Hz, PP = 1.00, PCC: expected value of group difference = 0.167 Hz, PP = 1.00). Furthermore, the self-inhibition of both MPFC (expected value of group difference = −0.119, PP = 1.00) and ACC (expected value of group difference = -0.217, PP = 1.00) was decreased in patients. A LOOCV within the PEB framework showed that patients were identified significantly better than random classification [area under the curve, AUC = 0.76, 95% CI (0.62 0.86), **Figure 2**]. When performing an LOOCV for the individual parameters, the ACC self-connection [AUC = 0.73, 95% CI (0.59 0.84)] was a significant predictor for diagnostic status.

### 3.4 Connectivity for selective serotonin reuptake inhibitors

An exploratory analysis of effects of SSRI intake revealed that the self-inhibition of both nodes of the DMN increased (MPFC: expected value of difference = 0.128 Hz, PP = 1.00; PCC: expected value of difference = 0.166 Hz, PP = 1.00) whereas self-inhibition of the ACC in the SN decreased (expected value of difference = -0.175 Hz, PP = 1.00, **Figure 1C**). The lower this parameter, the more readily the region is excited by the network inputs, i.e., patients receiving SSRIs during the study had increased input sensitivity in the ACC but decreased sensitivity in the DMN.

## 4 Discussion

The aim of this study was to compare brain connectivity of adolescents with MDD and matched healthy controls using spDCM during resting state. In our study cohort comprising 30 MDD patients, we conducted our analysis with a subset of 29 participants (excluding one patient), along with 32 healthy controls, as we examined the resting-state effective connectivity between principal nodes of the DMN and SN. Consistent with the growing literature that MDD is associated with a dysfunction of interactions of large-scale networks (46), our results indicate that altered effective connectivity within and between DMN and SN is a core feature of adolescent MDD. A leave-one-out cross-validation analysis showed that the effect size of DMN-SN interactions is sufficiently large to provide higher than chance prediction of diagnostic status in patients and healthy controls.

Corroborating our first hypothesis, we found that patients showed consistent weaker inhibition between the SN – particularly bilateral amygdalae – and the two principal nodes of the DMN (MPFC, PCC). This finding is in agreement with previous studies that report increased connectivity between the SN and the DMN in adult (9–14) and adolescent MDD patients (11, 18–22). Dysregulation of the affective brain during rest (i.e., weaker inhibitory bottom-up connectivity from SN to DMN) has been suggested to lead to excessive “emotional coloring” of thoughts and to symptoms such as rumination, negative affect, and an excessive self-focus (19). This is in line with the idea that altered large-scale network connectivity between the DMN and the SN is associated with maladaptive self-referential processing and emotional regulation in the triple-network model (29). Moreover, it has been suggested that depression is associated with differential integration of salience or precision signals (i.e., attentional control in the terms of predictive brain) in the brain (14). In particular, the amygdala is thought to carry information related to uncertainty about the predicted sensory input to the cortex (28, 31, 47, 48). In accordance with predicitive processing theory, these results could therefore indicate that amygdala-DMN dysregulation reflect a failure to estimate the precision for incoming sensory data for allostatic regulation and thereby sustaining depressive symptoms (24). Although research is only beginning to unveil the underlying neurobiological mechanisms of predictive processing, our findings suggest that changes in amygdala-DMN connectivity play a pivotal role in adolescent depression in accordance with earlier work (11, 18, 19).

Second, our results show that patients had a weaker self-inhibition of the ACC. Reduced self-inhibition might be interpreted as loss of precision. The self-inhibition parameter indicates how strongly a region inhibits its own activity when it receives inputs from other regions. In conjunction with the overall inhibitory connection from the rAMY to the ACC, this is consistent with a previous functional connectivity study conducted by Pannekoek et al. (21), who reported increased negative connectivity between the rAMY and ACC. The ACC plays a pivotal role in visceromotor control, serving as a hub that can initiate appropriate actions when the brain detects sensory prediction errors arising from either external stimuli or the internal milieu (28). In this context, the stronger inhibition observed from the amygdala to the ACC in patients might be linked to altered processing dynamics of behavioral control. Specifically, a heightened inhibition could impede the ACC’s capacity to effectively detect and respond to prediction errors, which are crucial for guiding adaptive behavioral adjustments. Besides the ACC, the MPFC also showed an increase in excitability in patients, exacerbating aberrant bottom-up influences from the amygdalae on its function.

The predictive coding theory posits that the excitatory-inhibitory balance primarily moderated by neuromodulatory systems and GABAergic interneurons governs the the precision of prediction errors in the superficial pyramidal neurons within a hierarchical cortical network (6, 24). Prediction errors are signals that measure the mismatch between priors on upper levels and sensed information on lower levels, which are thought to be crucial for learning and updating internal models of the environment. By modulating the gain or excitability of superficial pyramidal cells within a region, self-connections modulate the precision of the prediction error. A loss of synaptic gain control in a given area could reduce the precision of information encoded within a region, and diminish its influence over lower level areas. In our model, this would correspond to a loss of influence (i.e., precision) of the more abstract priors in cortical high level areas (MPFC and ACC) over the more concrete bottom-up sensory data. Together with weaker amygdalar inhibition of the DMN, we suspect that imprecise prior beliefs will shift the weight in cortical updating to ascending autonomic information. In terms of this framework, this might represent a critical loss of precise encoding of uncertainty und would entail a model of the world that looks less predictable and more surprising – a take with remarkable parallels to the learned helplessness theory (49).

Interestingly, for patients who received SSRIs, self-inhibition in the ACC was further decreased compared to patients who did not receive SSRI treatment. This altered synaptic gain by SSRIs is believed to represent a mechanism by which SSRIs can improve clinical outcomes (50). By enhancing the excitability or sensitivity of the ACC to external input, such as from the amygdala, SSRIs may facilitate the learning and updating of internal models of the environment, and promote the resolution of uncertainties, facilitating the regaining of control over internal beliefs and reducing the weight on bottom-up signals. Moreover, patients who received SSRIs had comparable levels of self-inhibition in the MPFC to healthy controls, in contrast to patients who did not receive SSRI treatment. This observation may indicate restored synaptic gain and increased network efficiency in MDD patients, potentially aiding in regaining control over precision estimates of internal beliefs. A recent study of brain connectivity in adolescent MDD reported that SSRI treatment responders have a distinct connectivity profile compared to healthy controls and non-responders (11). Specifically, they exhibited greater DMN-SN inhibition (MPFC, ACC) and greater within-SN inhibition (amygdala, ACC), which appeared to facilitate the response to SSRI treatment. These findings suggest that brain connectivity could serve as a valuable marker for predicting and monitoring treatment response in MDD. However, the precise mechanisms and implications of SSRI-induced changes in brain connectivity remain unclear and warrant further investigation.

Given the complex interactions between the multiple neurotransmitter systems (e.g., serotonin, dopamine, GABA, glutamate) on the synaptic level, it is impossible to disentangle individual contributions with spDCM that investigates neuronal ensembles. Nevertheless, several studies suggest reduction of GABA levels in the prefrontal cortex signalling in adolescent (51) and adult depression (52, 53). Animal studies show that the modulation of GABAergic interneurons can reestablish the excitation-inhibition balance (54) and that chronic SSRI treatment can stimulate the neurogenesis of GABAergic interneurons (55). Further evidence for this interpretation stems from the growing literature of ketamine in depression, that implicate the ACC as key target for the mood enhancing effect (56). Although the exact mechanism of antidepressant effect of ketamine remains unknown to date, it has been suggested that the blocking of NMDA receptor on GABA interneurons – and thereby attenuating GABA inhibition, which in turn leads to activation of pyramidal cells and promotes the release of brain-derived neurotrophic factor – might be critical for the alleviation of symptoms in adults (57) and adolescents (58). Yet, scrutinizing the individual contributions of neurotransmitters remains a difficult challenge, because of the intricate interactions on the synaptic level and multiple receptor subtypes expressed on GABAergic interneurons.

This study reveals new insights into the intrinsic brain connectivity in adolescents with MDD, however, it is not without limitations. Although our sample size is rather common for neuroimaging studies, it also reflects the recruitment challenges for this particular patient group. We used cross-validation procedures in our analysis to ensure the generalizability of results, nevertheless, future studies should replicate our results in larger cohorts to enhance the robustness of our findings and allow for more nuanced analyses. Second, to confirm clinical utility of these connectivity-based measures, longitudinal studies are required to study the change of symptoms alongside with changes in connectivity. The aforementioned study by (11) was the first to follow adolescent MDD patients to investigate treatment effects on effective connectivity longitudinally. Still, more studies that assess brain connectivity in parallel with treatment multiple times will be needed to understand the trajectories of symptoms in relation to neurobiological changes. Furthermore, in this study we focussed on the interactions between the DMN and the SN. Previous network models also implicate other large-scale networks (e.g. the cognitive control network, CCN, or reward network, RN) in depression (46). Understanding how neurodevelopmental trajectories between the DMN, SN, CCN, or RN affect mood will be pivotal for a comprehensive model of the disorder. Lastly, the scope of our study did not extend to the assessment of counseling interventions, which are distinct from psychotherapy in both accessibility and methodology. Future research should aim to include these interventions to better understand their potential in enhancing mental health outcomes.

In conclusion, this study sheds new light on the neurobiology of mechanisms in adolescent depression. We highlight the importance of the effective connectivity between – and within – DMN and SN during resting state in adolescent MDD. This connectivity pattern might represent a potential neurobiological marker of adolescent MDD and may be used to measure and predict depression. Our results suggest a new direction for studying mental health problems in adolescents and their respective treatments.

## Data availability statement

All relevant anonymised data and codes used to generate results are available from the authors on request, subject to compliance with the requirements of the ethics committee of the Canton of Zurich, Switzerland.

## Ethics statement

The studies involving humans were approved by Ethics comittee of the Canton of Zurich, Switzerland. The studies were conducted in accordance with the local legislation and institutional requirements. Written informed consent for participation in this study was provided by the participants’ legal guardians/next of kin.

## Author contributions

DW: Writing – original draft, Software, Methodology, Investigation, Formal Analysis, Data curation, Conceptualization. IH: Writing – review & editing, Investigation. II: Writing – review & editing, Software, Methodology. GB: Writing – review & editing, Resources, Investigation, Funding acquisition. SW: Writing – review & editing, Resources, Methodology. SB: Writing – original draft, Supervision, Project administration, Methodology, Funding acquisition, Formal Analysis, Conceptualization.
